# Assessment of Health System Readiness and Quality of Dementia Services in Peru: Protocol for a Qualitative Study With Stakeholder Interviews and Documentation Review

**DOI:** 10.2196/60296

**Published:** 2025-03-21

**Authors:** Maria Lazo-Porras, Francisco Jose Tateishi-Serruto, Christopher Butler, María Sofía Cuba-Fuentes, Daniela Rossini-Vilchez, Silvana Perez-Leon, Miriam Lúcar-Flores, J Jaime Miranda, Antonio Bernabe-Ortiz, Francisco Diez-Canseco, Graham Moore, Filipa Landeiro, Maria Kathia Cardenas, Juan Carlos Vera Tudela, Lee White, Rafael A Calvo, William Whiteley, Jemma Hawkins, IMPACT Salud Study Group

**Affiliations:** 1 CRONICAS Center of Excellence in Chronic Diseases Universidad Peruana Cayetano Heredia Lima Peru; 2 Department of Brain Sciences Imperial College London London United Kingdom; 3 The George Institute for Global Health UK London United Kingdom; 4 Center for Research in Primary Health Care Universidad Peruana Cayetano Heredia Lima Peru; 5 School of Social Sciences Centre for Development, Evaluation Complexity and Implementation in Public Health Improvement (DECIPHer) Cardiff University Cardiff United Kingdom; 6 Health Economics Research Centre University of Oxford Oxford United Kingdom; 7 Dyson School of Design Engineering Imperial College London London United Kingdom; 8 Centre for Clinical Brain Sciences University of Edinburgh Edinburgh United Kingdom; 9 See Authors' Contributions

**Keywords:** dementia, health system readiness, caregiver, comorbidities, Peru, study protocol, quality of care, comorbidity, patient journey, mHealth

## Abstract

**Background:**

Dementia is a global health priority with significant challenges due to its complex nature and increasing prevalence. Health systems worldwide struggle to address chronic conditions like dementia, often providing fragmented care. However, information about how health systems respond to the needs of people with dementia and their carers, and the quality of care provided, is scarce in low- and middle-income countries.

**Objective:**

This study aims to assess the quality of the health system to provide diagnosis and care for people with dementia and their carers in Peru. In order to do this, the study will explore the response of the Peruvian health system to people with dementia and their carers, and explore the experiences of people with dementia of receiving their diagnosis, management, and quality of care for this condition.

**Methods:**

This study is part of a research program called “IMPACT Salud: Innovations using Mhealth for people with dementia and Co-morbidities,” aimed at strengthening health systems to provide care for people with dementia and their carers. The study has a descriptive, cross-sectional design that uses a qualitative methodology, including stakeholder interviews and documentation review, and consists of 2 substudies, a health system assessment (HSA) and an exploration of the patient journey. The first substudy uses an HSA methodology suitable for low- and middle-income countries, conducting 160 structured interviews with 12 different stakeholder types across 3 levels of the health system (micro, meso, and macro) in 4 Peruvian regions, each with distinct geographical and urbanization profiles. The second substudy uses a patient journey methodology, which involves conducting 40 in-depth interviews with people with dementia, carers, and health care workers from the same 4 regions. The insights into the people with dementia patient and caregiver experience within the health system from the interviews will be used to produce a patient journey map. The analysis will be guided by the high-quality health system framework, and the findings from the HSA and patient journey will be structured using the domains included in the framework through the lens of quality of services.

**Results:**

Data collection began in March 2024. As of the end of September 2024, a total of 156 interviews from the HSA and 38 interviews from the patient journey study have been conducted across 4 regions.

**Conclusions:**

This study will provide a national, multilevel insight into the current operation of the Peruvian health system, including an analysis of the quality of services provided with regard to dementia diagnosis, management, and care from the perspectives of stakeholders, patients, and their carers.

**International Registered Report Identifier (IRRID):**

DERR1-10.2196/60296

## Introduction

### Background

Health systems worldwide face the challenge of addressing chronic conditions, which in low- and middle-income countries (LMICs) is exacerbated due to resource scarcity. LMICs often respond to diseases in isolation through vertical programs [1]. A global and integrated response to chronic conditions is needed to provide high-quality care for individuals with complex conditions [2]. Dementia is one such chronic condition, characterized as a complex disorder involving psychosocial dysfunction and vulnerability due to brain disease [3]. Dementia is a generic term that describes progressive cognitive and behavioral decline severe enough to interfere with daily life and independent function [4] and is a global health priority given its enormous human and economic costs.

Globally, the number of people with dementia is increasing. According to the World Health Organization, there are approximately 50 million people with dementia, projected to reach 75 million by 2030 [4]. Studies indicate that women are more likely to develop dementia than men [5]. Furthermore, around 60% of people with dementia live in LMICs, which are aging rapidly and have limited capacity to support them [5]. In Peru, in 2022, the Ministry of Health (MoH) attended to 13,066 people with Alzheimer disease and other dementias [6].

Health systems in LMICs are not well equipped to address dementia, often resulting in inadequate or nonexistent care. Specific challenges for the health systems and people with dementia include developing effective diagnostic services, managing cognitive and behavioral decline, handling multiple comorbidities, caregiver burden, widespread stigma, and lack of awareness about dementia. The concept of tracer conditions can help facilitate understanding the complexity of health systems and the challenges LMICs face [7,8]. Using tracers in health systems research is based on the premise that focusing on carefully selected health problems allows for the identification of weaknesses within the system and facilitates more direct insight of its performance [7,8].

Dementia affects multiple aspects of individual and family well-being, serving as an indicator of multimorbidity in both people with dementia and their carers. People with dementia have twice the number of chronic physical and mental conditions compared to those without dementia [9,10]. Dementia shares risk factors with many other common chronic diseases, including health-related behaviors (eg, unhealthy diet, smoking, and physical inactivity), as well as obesity, hypertension, diabetes, depression, and social isolation. Together, these factors account for 35% or more of the population-attributable risk (proportion of new cases of dementia due to the noted exposures). In Latin America and the Caribbean, this percentage can reach 56%, due to a combination of cultural, political, and economic factors [11].

In a broader context, addressing dementia does not mean working with a disease in isolation but rather a broader set of system-wide responses, both within and outside health care delivery structures, which can in turn contribute to achieving global goals such as Universal Health Coverage and the Sustainable Development Goals [12-16]. Abundant literature has pointed out significant deficiencies in protection systems that are relevant for addressing dementia. In Peru, despite having a specific law that addresses dementia (law no. 30795: Law for the Prevention and Treatment of Alzheimer's Disease and Other Dementias) [17], people with dementia are affected by the fragmented health care systems, shortage of human resources, limited specialized services, minimal or nonexistent long-term care, and a siloed approach to addressing diseases and health conditions.

In the vast majority of cases [18-22], support available for individuals with chronic conditions in Peru, including dementia, comes from family members, predominantly women [23], who commonly lack access to information and primary or specialized dementia care services. A study across various LMICs identified high psychological stress and caregiver burden, particularly among female carers of people with dementia [24]. Studies, such as those by Papastavrou, recognize caregiving for people with dementia as highly stressful for families, potentially leading to depression, panic disorder, anxiety, or substance use like psychotropic drugs, alcohol, and nicotine.

To date, few studies have addressed dementia in South America. Notably, the 2023 Pan American Health Organization report [25] indicates that only Chile has a national dementia plan, with underreporting in LMICs potentially reaching 90%. The 10/66 Dementia Research Group study [25] highlights the high cost of dementia for health systems and the negative mental health impacts on women in LMICs, who are often primary caregivers for people with dementia [26]. This underscores the need for further research on dementia diagnosis and care in LMICs and for directing it effectively toward impactful decision-making. This landscape highlights the urgent need for improved dementia diagnosis strategies in Peru and LMICs, considering the unique challenges posed by limited resources, illiteracy rates, and caregiver burden. Task-shifting approaches recommended by the World Health Organization could help bridge the gap in dementia care [4]. Additionally, addressing the social and economic impacts of dementia requires a multifaceted approach, involving collaboration between health care providers, policy makers, and community organizations. To understand the potential for such improvements on dementia diagnosis and care in Peru, a thorough exploration is needed of the existing health system and the problems that people with dementia and their carers face in daily life from a multistakeholder perspective. For these reasons, this study aims to assess the readiness to diagnose and treat dementia, as well as the quality of dementia services, in the Peruvian health system from the perspective of various stakeholders.

The study has 2 overarching objectives, which are addressed by 2 parallel substudies, respectively: a health system assessment (HSA) and an exploration of the patient journey within the health system.

### Substudy 1: HSA

The HSA aims to understand the diagnosis and treatment needs of people with dementia and their carers in Peru and resources available for this, including the readiness of the health system to implement innovative mobile health (mHealth) tools for the screening and diagnosis of dementia. It will address the following research questions (Textbox 1).

**Research questions.**

- What is the existing capacity of health workers to provide care to people with dementia and what is the quality of this care?
- What are the preconceived barriers and enablers for engaging with interventions using mobile health (mHealth) technology?
- What are the main system barriers and facilitators for the uptake of mHealth tools for supporting people with dementia and carer dyads?

### Substudy 2: Patient Journey

This substudy aims to understand the journey of people with dementia to being diagnosed and receiving management for dementia and to identify opportunities to improve the diagnosis and management of dementia for people with dementia. It will address the following research questions (Textbox 2).

**Research questions.**

- What are the experiences and challenges of providing care and unmet needs of people with dementia, their carers, and health workers?
- What are the most common barriers and facilitators to being diagnosed with dementia from the perspective of people with dementia, their carers, and health workers?
- What are the most common barriers and facilitators to receive or provide treatment and management of dementia from the perspective of people with dementia, their carers, and health workers?

## Methods

### Context

The IMPACT Salud research program comprises 4 distinct work packages (see Table 1), which have the overarching goal of strengthening the health system in Peru through sustainable, integrated, community-delivered, technology-enabled innovations. The expected outcomes of the overall program include improving access to diagnosis of dementia and development of a feasible and acceptable mHealth intervention for people with dementia and their carers. The first work package, the focus of this protocol manuscript, is a descriptive cross-sectional qualitative study, consisting of an HSA and an exploration of the patient journey through interviews and documentation review in order to inform future work packages of the program. The HSA will collect information on the structure and organization of the Peruvian health system, the political environment, financing, data collection and information systems, availability and affordability of medications and tests, barriers to diagnosis and treatment, service delivery in prevention and management issues, training and capacity to provide care, medical technologies and infrastructure, and perceptions of and experience with using mHealth technology.

**Table 1:** Work packages that comprise IMPACT Salud.

Work packages	Objective
1	Evaluate health system readiness to diagnose, treat, and support people with dementia and carers
2	Develop and implement a mobile health–enabled system for the diagnosis of dementia
3	Determine the feasibility of an intervention to treat and support people with dementia and their carers
4	Assess the economic burden of dementia and related comorbidities in Peru and estimate the costs of rolling out the diagnosis tool at a national level

This information will be collected from diverse stakeholders from different institutions, including not only health services provision, eg, civil society and municipalities, among others, but also from policy and practice documentation. The patient journey study will complement the information gathered from the HSA from a more centered perspective of people with dementia and their carers. The insights into the experiences of both people with dementia and carers within the health system will inform the development of a journey map that visualizes these experiences. The findings from the HSA and patient journey will inform other work packages of the program and will allow for the identification of opportunities for using mHealth technology for dementia diagnosis improvement and management intervention.

### Setting

#### Study Regions

The IMPACT Salud program is working across 4 sites in Peru (see Table 2). These include Lima, the nation's capital with over 10 million inhabitants [27], situated along the central coast. Lima holds significant importance as the country's primary city, burdened with the highest incidence of noncommunicable diseases [28]. Notable for its social disparity and cultural heterogeneity, Lima is a dynamic research locale. The second city, Huancayo, has over half a million residents [29] and is positioned in the central highlands at an elevation of 3200 meters above sea level. A pivotal hub for the economic growth of the central region, Huancayo attracts migrants from the jungle and southern highlands, primarily engaged in providing diverse services for citizens and agricultural activities. The third city, Iquitos, serves as the primary urban center in the jungle, hosting almost half a million inhabitants [30]. Characterized by diverse indigenous populations, Iquitos can only be accessed via river and air transport, resulting in an economy marked by high transportation costs. Lastly, Tumbes, located in Peru's northern most region with 265,844 residents [31], operates as a border economy due to its proximity to Ecuador. With a warm, rainy climate, Tumbes is currently experiencing heavy rainfall attributed to the “El Niño-Southern Oscillation” climatic phenomenon.

**Table 2:** Characteristics of the study sites or locations. Source: Instituto Nacional de Estadística e Informática (INEI)-2017—Repositorio Único Nacional de Información en Salud (REUNIS) [27-30].

Sites	Population, n	Region	Illiteracy rate in the department (15+), %	Completed high school in the department (15+), %	Most spoken language in the department	Quintile of dementia care in Peru
Lima (Metropolitan)	8,894,412	Coast	2	50.2	Spanish	1
Huancayo	545,615	Highlands (Andes)	5.3	45.5	Spanish	3
Iquitos—Maynas	149,773	Amazon Jungle	5.4	50.1	Spanish	1
Tumbes	2,154,962	Coast	4.1	48.3	Spanish	4

#### Health Care System

The Peruvian health care system is fragmented and complex, with health care provision and financing depending on multiple public and private entities under the oversight of the MoH. Within this fragmentation, the 2 main public health care providers are the MoH and the Social Security System (EsSalud), the last one depending financially on the Ministry of Labour and Employment Promotion [32].

This study will primarily work with the MoH, which serves 74.5% of the population [33]. Since 2007, there has been a coordinated and decentralized health system [34], which has resulted in the creation of regional health directorates and management for each region that, independently, are responsible for implementing MoH's regulations.

For the purpose of this research, we categorize the health system into 3 levels: micro, meso, and macro. The micro level encompasses primary health care services, along with any supplementary systems or organizations at the local level. The meso level includes secondary health care services and regional directorates. Finally, the macro level encompasses tertiary health care services, national directorates of the MoH, and specialized bodies.

### Design

The HSA follows a Rapid Assessment Protocol (RAPIA) methodology for data collection, targeting access to care for individuals with chronic diseases in LMICs [35]. The RAPIA framework is being used for its structured approach to data collection, designed to generate information from both primary and secondary sources, including structured interviews with participants across various levels of the health system within the 4 representative regions. The interview questions were adapted to gather information on dementia, modifying the focus on medications and emphasizing inquiries about the care provided to people with dementia by their caregivers. Key attributes of this methodology include its patient-centric focus, cost-effectiveness, and provision of valuable insights for decision-makers. The rapid assessment methodology is implemented via structured interviews following a standardized questionnaire, supplemented with a review of secondary sources of information (eg, policies and national statistics), to explore 11 themes related to the Peruvian health system across the 3 levels of the health system (macro, meso, and micro). This framework will facilitate the understanding of the needs and available resources for patients with dementia and their carers. Similarly, to understand the experiences of people with dementia in receiving their diagnosis and the subsequent management of their condition, we will use the “patient journey” methodology. This approach is used by health care managers to identify gaps in the touchpoints between the health care system and the patient throughout the care process, such as the admission process, physical care, and appointment reminders [36]. In LMICs, “patient journeys” for noncommunicable diseases provide valuable insights for decision makers aiming to prioritize interventions and optimize disease management [36]. To carry out the “patient journey,” we will conduct in-depth qualitative interviews with individuals with dementia, their caregivers, and health care professionals. This follows an example of a study on understanding poststroke care management [37]. Based on the patient journey interviews, a patient journey map will be developed to visualize the experience of people with dementia, carers, and health care workers within the health care system.

### Participants and Selection Criteria

#### Overview

Table 3 details the different types of participants across the 2 substudies and the criteria used for purposive selection of participants. In the HSA, 14 types of key actors are participating across the macro [4], meso [3], and micro [7] levels. The patient journey involves 3 types of key actors: people with dementia, their carers, and health care workers. The aim is to achieve a comprehensive coverage of health care system stakeholders pertinent to the theme of dementia.

**Table 3:** Key actors and inclusion criteria.

Level			Target institution and key stakeholder role		Inclusion criteria
**Substudy 1**					
	Macro	(1) Ministry of Health (eg, Mental Health Strategy, Non-Communicable Diseases Strategy, Integrated Health Insurance (SIS) Department, Planning and resources) (2) Ministry of Financing (eg, Presupuesto por Resultados) (3) Social Security (EsSalud) (4) Ministry of Women and Vulnerable Populations (5) Key opinion leaders (eg, nongovernmental organization representatives, representatives of health organizations)		18 years of age or older Occupy the specified role in the Peruvian provinces of Lima, Huancayo, Iquitos, or Tumbes	
	Meso	(6) Regional directorates of health (7) Health workers of mental health public hospitals, general hospitals, and specialized memory clinics (eg, including psychologists and therapists) (8) Nursing homes run by District Municipalities		18 years of age or older Occupy the specified role in the Peruvian provinces of Lima, Huancayo, Iquitos, or Tumbes	
	Micro	(9) Grass-root organizations and elderly community centers (Centro del Adulto Mayor) (10) Community leaders and community health workers (11) Health care workers (eg, physician, nurses, technicians, pharmacies) from primary health care facilities and community primary mental health care facilities (Centro de Salud Mental Comunitario)		18 years of age or older Occupy the specified role in the Peruvian provinces of Lima, Huancayo, Iquitos, or Tumbes	
	Micro	(12) People with dementia and carers		Only for people with dementia; people with a mild dementia diagnosis who meet the following criteria: Status of dementia: people will be asked for a self-reported diagnosis of dementia. Where this is not possible, the carer will be asked to confirm. This diagnosis had to be performed by a physician in a health facility. Level of functionality: the Pfeffer Functional Activities Questionnaire (PFAQ), Spanish version, will be applied with potential participants (n=31); participants must score below 6 to participate. People with dementia should also have at least one chronic comorbidity, such as hypertension, diabetes, depression, and anxiety, among others. Carers: people who are formal or informal carers, including family members that are responsible for taking care of the people with dementia. They need to have been with the people with dementia in the process of diagnosis and management and are self-recognized as carers of the people with dementia. We will also include carers of people with severe stages of dementia	
**Substudy 2**					
	N/A^a^	(1) People with dementia		Same as substudy 1	
	N/A	(2) Carers		Same as substudy 1	
	N/A	(3) Health care workers		General health practitioners and neurologists	

^a^N/A: not applicable.

#### Sampling Method

Across the HSA and patient journey substudies, we aim to include 200 participants in total across the 4 sites through purposive sampling to recruit the required range of stakeholders across the health system. The inclusion criteria are detailed in Table 3, and the target sample size is provided in Table 4. These numbers will allow for gathering sufficient information from all regions to understand differences across the 4 settings and levels. For recruitment of people with dementia, there are specific inclusion criteria related to the stage of dementia and level of functionality. A community mapping exercise was conducted in each region to identify relevant organizations and groups from which to recruit potential participants. The purposive sampling was focused on identifying participants who represented each stakeholder type across each level of the health system as outlined in Table 4 and continued until all stakeholder types had been recruited.

**Table 4:** Sample size.

		Huancayo	Iquitos	Lima	Tumbes	Total
**Substudy 1**						
	Macro^a^	—	—	—	—	20
	Meso	15	15	15	15	60
	Micro	20	20	20	20	80
**Substudy 2**						
	People with dementia and carers	6	6	6	6	24
	Health care workers	4	4	4	4	16

^a^The precise breakdown of participants is unclear for the 4 provinces.

### Fieldwork Team

Field workers in each site were selected for their expertise in conducting qualitative interviews, their health care sector experience, and residency in the designated city of work. A total of 11 field workers have been engaged, with 9 assigned to micro- and meso-level interviews and 2 for the macro-level. A dedicated team of 2 or 3 researchers has been formed for each site, alongside a coordinator. Fieldwork team members attended 2 comprehensive training sessions, combining in-person and virtual formats. Training topics encompassed participant recruitment protocols, ethical considerations in elderly populations and dementia care, implementation of informed consent and assent procedures, an introduction to dementia from biomedical perspectives, and familiarization with the data collection materials. Upon completion of training, field workers received an operational manual detailing standardized operating procedures for data collection.

### Data Collection Procedures

**Figure 1:**
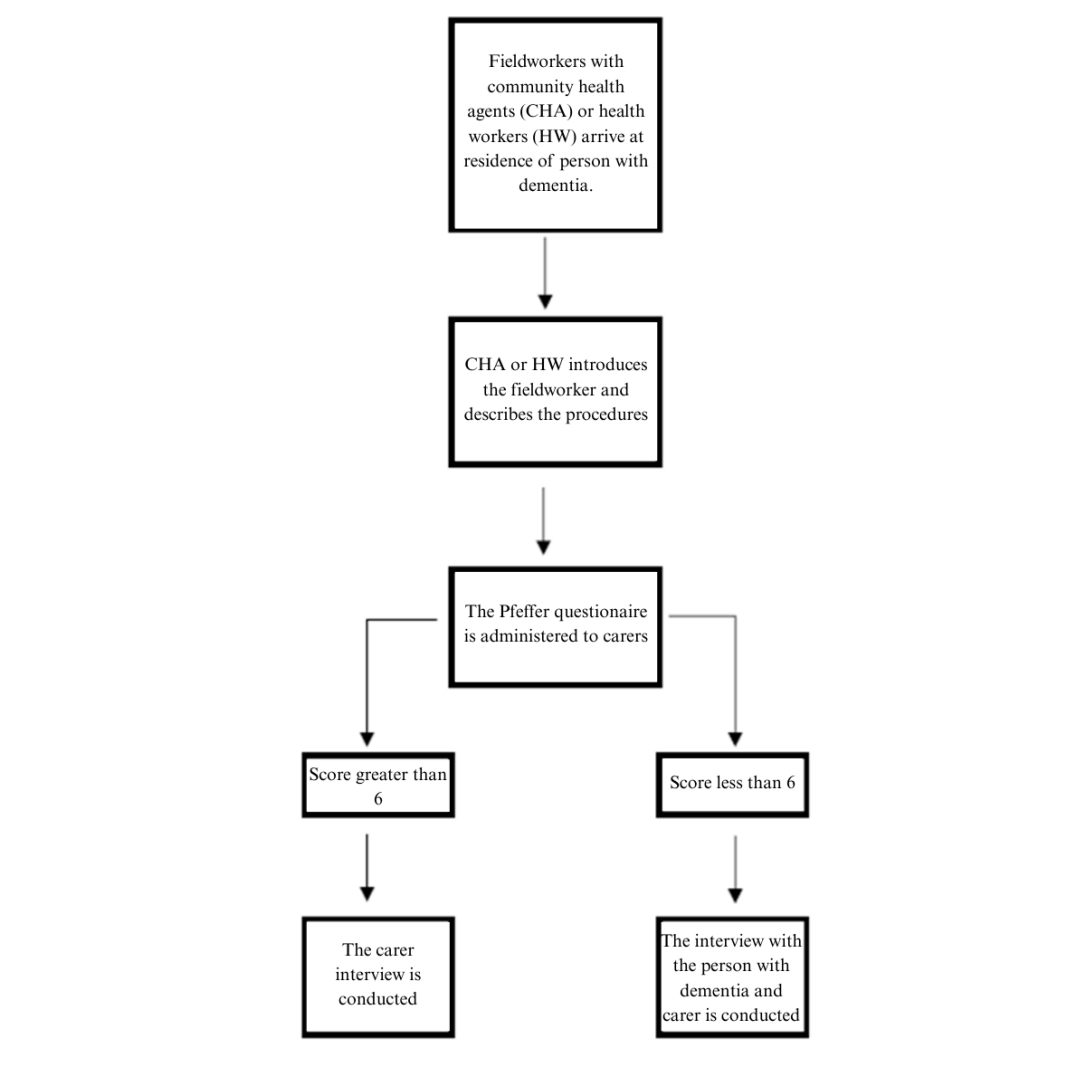
Procedure for conducting interviews with people with dementia.

The study received institutional support from MoH and follows a staggered data collection approach. The process commences with higher management levels progressively authorizing interviews at operational tiers such as first-level health centers. Identification of persons with dementia (diagnosed by a health center) and their carers is facilitated through community health agents, health care center workers, and health care facility administrations. They introduce the fieldwork staff, who then administer the Pfeffer Functional Activities Questionnaire [38,39] to assess the people with dementia's functional abilities for daily activities and their possibility to participate in the interview. Scores below 6, indicating no clear impairment of functional activities, prompt interviews with both the caregiver and the person with dementia. In such cases, an informed assent is sought from the person with dementia, with informed consent obtained from the carer in their representative capacity. Figure 1 summarizes the procedure for conducting interviews with people with dementia. For other actors, specific informed consent procedures are followed prior to initiating data collection. Detailed information regarding recruitment can be found in Multimedia Appendix 1.

For the HSA, interview information will be recorded in notes on a printed version of the interview questionnaire and captured with handheld recorders. These notes will then be entered into the REDCap (Research Electronic Data Capture; Vanderbilt University) software, developed by Vanderbilt University. In this case, interviews will not be transcribed; only the recordings will be used as backup. This is due to the format of the RAPIA methodology, which advocates for a rapid analysis of information, prioritizing the perspectives of various actors rather than delving deeply into them; hence, a detailed analysis of the testimony is not necessary. On the other hand, for the patient journey, the interviews will be recorded and subsequently transcribed verbatim and coded in an Excel spreadsheet. Following coding, the information will be shared in order to seek its validation at a meeting with people with dementia and careers who were interviewed.

### Data Collection Materials

Questionnaires with closed and open-ended questions will be used to perform the structured interviews conducted in the HSA. These instruments were originally designed to assess health systems with regard to access to insulin [35]; subsequently, they have been applied for exploring chronic diseases and neglected tropical diseases [1]. Drawing from these previous experiences, they have been adapted for this study, with questions amended or added to capture information on several key thematic areas to assess the readiness of the Peruvian health system for diagnosing and managing dementia. These thematic areas are informed by the RAPIA guidelines mentioned earlier and are outlined in more detail in Table 5, including sections and topics of the structured interview questionnaire; an example of the structured questionnaire is also provided in Multimedia Appendix 2. As per the RAPIA guidelines, interview data will be supplemented with a document review of relevant technical standards, laws, regulations, national plans, care protocols, clinical guidelines, and other materials that are publicly available. In the patient journey, we will use semistructured interview guidelines for the in-depth interviews with people with dementia, carers, and health care workers. The guidelines will explore topics to understand the diagnosis and management process of dementia and identify opportunities for improvement. The interview guide is structured into 3 stages: prediagnosis, diagnosis, and treatment [40]; an example is provided in Multimedia Appendix 3. In these stages, the number of interactions with health care personnel, the time between appointments, the emotions associated with the encounters, among others, will be collected.

**Table 5:** Sections and topics of the questionnaire in the health system assessment.

Type of data	Components
General information	Place and date Contact information Demographic information Workplace and time Occupational category
Health system structure and organization	Units or departments responsible for people with dementia Preparation of health personnel Preparation to attend comorbidities Services and organizations centered in people with dementia
Relevant policies	Public policies and relevant policies for people with dementia
Financial issues	Sufficient funding for prevention, diagnosis, and treatment in people with dementia Barriers or difficulties for find funding for people with dementia Programs or financial contributions for people with dementia and careers
Data collection and information systems	Date registration for people with dementia Statistic information available about people with dementia and comorbidities
Service delivery in prevention and management	Knowledge of the available tests for people with dementia Medications for people with dementia Peruvian political medication for people with dementia Knowledge of the available medication for people with dementia Barriers or difficulties in the access of medication, tests and technology for people with dementia
Barriers for diagnosis	Current barriers to dementia assessment Current barriers to dementia attention Current barriers of dementia care
Training and capacity to provide care	Training, knowledge and capacity of health care workers in the provision and management of dementia care
Medical technology	Infrastructure to support internet access Infrastructure to support use of mHealth
Perceptions of and experience with using mHealth technology	Knowledge of the tools for the dementia diagnosis Opinion about mobile applications for disease diagnosis

### Analysis Procedure

The analysis of substudy 1 HSA will follow a deductive process using coding reliability thematic analysis [41], with the aid of a structured codebook to index the information collected within the domains proposed by the high-quality health system (HQHS) framework developed by Kruk. The HQHS framework was designed to take into account the challenges of LMICs and allows for a comprehensive evaluation of service quality beyond mere access, emphasizing patient-centered care and trust in health care systems. The framework will allow us to organize and structure the results of our substudies in order to create an overall overview of the health system and the quality of services provided to people with dementia and their carers. The relevance of using this framework lies in its ability to integrate general aspects, such as access to health care centers and the number of consultations, with the patient's perception of the service's utility when describing service quality. In contrast, the analysis of the patient journey in substudy 2 will be inductive, following a framework analysis approach [42]. Analyses for both substudies will be carried out in parallel until the final phase, at which point they will be integrated to enhance the HQHS framework. The detailed analytical procedures are outlined below.

Substudy 1 will follow a 3-step process, conducted initially by 2 researchers working together on data from one site first to agree on understanding of codes and then moving to work independently on interviews from the additional sites subsequent to this. Throughout the process, the team overseeing the study will meet regularly to discuss the coding of data into the various categories and thematic areas in order to confirm understandings and address any disagreements in data coding placement (Textbox 3).

Substudy 2 will consist of 4 phases (Textbox 4).

The findings will be reported in accordance with the checklist proposed by the COREQ (Consolidated Criteria for Reporting Qualitative Research) reporting guidelines for qualitative studies [43].

**Phases of the analysis for substudy 1.**

- First, the data from the structured interviews and document review will be coded into the 11 themes proposed by Rapid Assessment Protocol (RAPIA; Table 5), where each theme will be structured according to the interviewees and the regions they represent.
- Next, the data from stakeholders as coded into the RAPIA themes will be cross-referenced with the components and subcomponents of the high-quality health system framework.
- The final step will involve integrating relevant information from substudy 2 into this matrix.

**Phases of the analysis for substudy 2.**

- The first phase will involve developing an analytical framework based on codes derived from initial familiarization with the data. This familiarization process will consist of 4 meetings (one meeting per site) in which 3 researchers will listen to the interview transcripts and propose categories for analysis. The categories resulting from the first meeting will be used in the subsequent meetings, where their relevance will be evaluated.
- The second phase will involve coding all the data into the identified codes.
- The third phase will consist of summarizing data from the codes into overarching categories that represent key milestones identified as significant within the patient experience, as well as those identified by their carers and health care workers.
- These overarching category summaries will serve as the foundation for creating a patient journey map to visualize the data and for describing the subcomponents and components of the high-quality health system framework as identified in substudy 1.
- The draft patient journey map will be presented to a subsample of substudy 2 participants in each region to sense check the understanding of their experiences and inform a final version of the map and to feedback into the refinement of the overall analysis.

### Ethical Considerations

The research protocol, data collection materials, and consent were initially reviewed and approved by the institutional review board (IRB) at Universidad Peruana Cayetano Heredia (UPCH) on July 20, 2023 (IRB number 209080). Subsequent amendments were approved by UPCH on October 30, 2023, and May 13, 2024, and an extension was granted on June 18, 2024, allowing data collection until June 2025. The protocol and consent forms were also approved by the Imperial College Research Ethics Committee (IRB number 6784708) on February 29, 2024, with a further amendment approved on June 11, 2024. The study will be conducted in accordance with the recommendations for physicians involved in research on human subjects adopted by the 18th World Medical Assembly, Helsinki 1964, and later revisions. Participation in the interviews will be completely voluntary; potential participants will be provided with information about what taking part will involve, and signed consent will be sought for each participant prior to collecting any data.

All invited participants have the option to decline the invitation or withdraw from the study at any time. No monetary or material compensation has been provided, other than a brochure containing general information about dementia. All collected data will be anonymized prior to analysis, in order to protect confidentiality.

In addition to the considerations mentioned earlier, specific measures to ensure ethical safeguards and to abide by local legislation will be taken in the cases of people with dementia and their carers. Assent and dissent will be respected from people with dementia, verbally or nonverbally. Also, carers who function as proxy decision makers will also be consulted about consent when the potential participant has not been able to give full consent.

## Results

The IMPACT Salud program was funded in October of 2022, but the study was launched in Peru in November 2023. In the meantime, ethical approval and pilots were conducted before the implementation of both substudies. The pilot study took place from July to September 2023. The implementation of both substudies began in March 2024. As of September 30, 2024, 192 individuals have been interviewed as part of the HSA and the patient journey study from the cities of Iquitos, Huancayo, Tumbes, and Lima. Transcription and data systematization in REDCap will occur simultaneously with the administration of questionnaires and interviews. The analysis phase is scheduled to take place from October 2024 to January 2025.

## Discussion

### Principal Findings

This study, with its focus on both HSA and understanding the patient journey, will generate comprehensive insights into how dementia is diagnosed and managed across the diversity of the Peruvian health care system. The findings will be organized in 3 core themes from the HQHS framework, such as the process of care, quality impacts, and foundations. It is anticipated that this will reveal areas of strength in dementia care as well as areas where there are issues with quality of provision or lacking provision. This might include, for example, structural issues with financial resourcing, training of health care workers, or the existence of standardized policies and protocols. It is anticipated that there may be divergence between the experiences of stakeholders at different levels of the health system, as well as across the different regions in which the study is being conducted. Furthermore, the study will reveal insights into the facilitators and barriers to using mHealth technology in Peru for supporting better diagnosis and management of dementia.

To date, few studies have holistically addressed dementia in South America. This work aims to serve as the foundational step in a series of studies across LMICs, particularly in Latin America, assessing health care system readiness to effectively diagnose, manage and provide care for people experiencing dementia and chronic comorbidities. As the burden of dementia and associated conditions rises among older populations in LMICs [44-46], there is a dearth of data on health care system preparedness. Additionally, the study will also address the needs of other work packages as part of the IMPACT Salud program (see Table 1).

It is important to note that using health care system evaluations from high-income nations for public health decisions in LMICs is cautioned against due to considerable disparities in home care systems, health care professional readiness, belief and health literacy, technology and infrastructure availability, as well as the political stability essential for sustained quality care. As such, this research endeavors to provide updated insights into the intricate workings of health care systems in Peru, potentially driving reforms in the health care sector. Furthermore, it aims to foster alignment among stakeholders and amplify the impact of reforms on dementia patients' lives. A notable antecedent of health care systems' responses to dementia is the STRIDE project that engages 7 countries (Mexico, Brazil, India, Indonesia, Jamaica, Kenya, and South Africa) [47]. There is one publication of this study in Mexico, and STRIDE, like IMPACT Salud, involves actors from civil society, such as the Alzheimer's Association of Mexico, its allied associations, and researchers. However, STRIDE does not directly include the participation of patients and carers as project participants, as its goal is to improve the implementation of public policies rather than gathering the needs and experiences of people with dementia in the health care system [48]. A comparative analysis of findings between these projects is planned, aiming to elucidate differences and similarities. The results of this research also aim to provide input for the development of Comprehensive Care Guidelines for individuals with Alzheimer disease and other dementias, a task that the MoH has pending since its proposal in the 2018 regulation of the Law for the Prevention and Treatment of Alzheimer's Disease and Other Dementias [17].

Also, the study has a particular focus on carers due to the significant impact of caring for a person with dementia on their mental health. Carers are 4 times more likely to experience depression and 3 times more likely to experience anxiety [49,50]. For these reasons, it is important to explore the needs of carers and possible opportunities to better support them and reduce any burden they experience. The intervention that will be adapted [51] in work package 3 of the IMPACT Salud program (Table 1) will have carers as its focus, aiming to provide guidance and tools with the aim of improving the quality of life of people with dementia and the mental health and quality of life of their carers.

### Study Strengths and Limitations

The RAPIA methodology offers a diverse, multilevel, and national perspective to assess the health system, in this case having dementia as a tracer condition in Peru. While the patient journey complements this by providing insights from a patient-centered perspective about the health care system’s functionality.

The HQHS framework will enable the analysis to be organized to identify structural issues (such as access, political support, and availability of qualified health care personnel), as well as relative or subjective issues contingent upon each dementia patient, caregiver, and health care provider (such as trust and service quality).

The decision of using a combination of methodological approaches is based on their focus on the end user of the health system, namely persons with dementia and their carers. Furthermore, the RAPIA methodology has the quality of being flexible to the context, allowing the addition of topics to the interview and new questions to the questionnaire [35]. Finally, the methods offer replicability benefits for researchers and health system specialists in terms of time and costs to implement.

The approach proposed in this study involves examining the macro view while also taking into account the daily lives of people living with dementia. By staying focused on these, we aim to propose solutions in the health system that have a tangible impact on people's lives. The study includes 4 sites with diverse characteristics in terms of population size, ecosystem, mother tongue, and number of people with dementia attended by MINSA; this will allow comparing the quality of the health system and experiences of people with dementia and carers, as well as opportunities in 4 highly diverse contexts within Peru.

While the study has adopted existing robust methodologies for HSA and understanding patient experience, there are potential limitations with applying these in practice. To date, the study has faced difficulties in recruiting dementia patients who meet the inclusion criteria. Our data so far indicate that most patients seek diagnosis late, resulting in worse cognitive decline than expected (a score of less than 6 on the Pfeffer functional test). This issue is compounded by the view of dementia as a normal part of aging, which discourages symptomatic individuals from seeking health care. We have partially addressed this by conducting more interviews with carers in cases where patients themselves do not meet the inclusion criteria.

Furthermore, while the setting for the research includes 4 sites with diverse characteristics to represent the various sociodemographics and cultures of Peru, this is not exhaustive, and there may be factors related to the diagnosis and management of dementia that remain unidentified by this study.

Additionally, the HQHS framework has been used previously with a survey to share information from some domains or subdomains [52]. However, there is not much published research using this framework with qualitative data, so there may be some challenges faced with this during the analysis phase.

### Conclusions

This study will provide a national, multilevel insight into the current operation of the Peruvian health system, including an analysis of the quality of services provided. The findings will be structured in 3 core themes from the HQHS framework (process of care, quality impacts, and foundations) in order to share information about dementia diagnosis, management, and care from the perspectives of stakeholders, patients, and their carers.

### Dissemination

The dissemination strategy of the IMPACT Salud program is focused on sharing the study findings with a wide variety of audiences, including the patient and carer community, health care providers, and policy makers. A variety of dissemination formats are planned, including online and in-person meetings and webinars, policy briefs and briefing notes, social media, and news communication and scientific formats including peer-reviewed journals and conferences. We will also use the project web page [53] to share information for these different audiences in a friendly format.

## Appendix

Multimedia Appendix 1 Study manual detailing recruitment procedures.

Multimedia Appendix 2 HSA (health system assessment) questionnaire example - health workers and patient caregiver.

Multimedia Appendix 3 Patient Journey Interview Guide Example - Caregiver.

## Abbreviations

COREQ: Consolidated Criteria for Reporting Qualitative Research
HQHS: high-quality health system
HSA: health system assessment
IRB: institutional review board
LMICs: low- and middle-income countries
mHealth: mobile health
MoH: Ministry of Health
RAPIA: Rapid Assessment Protocol
REDCap: Research Electronic Data Capture
UPCH: Universidad Peruana Cayetano Heredia

## Data Availability

The datasets generated or analyzed during this study will initially be available in anonymized format from the study investigators (MLP, JH, SCF, and CB) upon reasonable request, in accordance with participants’ informed consent and IRB requirements. No publicly accessible repository is currently used for data sharing, but this will be developed as part of the IMPACT Salud study and more information will be available on the study website [53] in due course.
