# Supplementary material for: Assessment of Health System Readiness and Quality of Dementia Services in Peru: Protocol for a Qualitative Study With Stakeholder Interviews and Documentation Review
Source: JMIR Res Protoc. 2025 Mar 21;14:e60296. doi: 10.2196/60296 (PMC11971575; doi:10.2196/60296)
Supplement: Multimedia Appendix 1 [file resprot_v14i1e60296_app1.pdf]

OPERATIONAL MANUAL: FIELDWORK

This document described the recruitment procedures provided to the field workers to identify and invite participants to the study in the four regions.

I. SUB-STUDY 1 AND 2 HEALTH SYSTEM ASSESSMENT AND PATIENT JOURNEY MAPPING.

I.1. Recruitment Procedure

The same recruitment process will be followed for Sub-study 1 and Sub-study 2, as both data collection efforts are intended to take place simultaneously. The difference is that for Sub-study 2, "patient journey mapping," only patients with dementia, carers, and healthcare workers will be recruited

Sub-study 1 will begin with the selection of community mental health centers, health centers, and hospitals to recruit the desired participants. The first section details the criteria for selecting healthcare facilities, followed by a list of actors to be recruited along with their inclusion criteria. Sub-study 2 will recruit healthcare workers from the health centers selected in Sub-study 1

I.1.1. Selection of Community Mental Health Centers, Health Centers, and Hospitals

The criteria for selecting healthcare facilities will include the following:

1. The facilities will be located in the cities where the research will be conducted: Lima, Huancayo, Iquitos, and Tumbes.
2. They will be situated within the urban area of the city.
3. They will be classified as Community Mental Health Centers.
4. They will be health centers capable of referring dementia cases to Community Mental Health Centers.

Authorization for collecting information at healthcare facilities will be requested through the DIRESAS, GERESAS, or DIRIS. Each facility will be selected strategically and individually. Given that the number of interviews to be conducted at health centers will not cover all facilities meeting the selection criteria, and due to insufficient information regarding the dementia experience of each center and healthcare professional, authorization will be sought from a number of centers exceeding the project's capacity. The strategy will involve mapping the dementia experiences of each health center through discussions with their directors. This

information will be used to design a sample that meets the proposed quotas and captures the heterogeneity of perceptions regarding dementia issues.

During the mapping process at the Community Mental Health Centers (CSMC) and health centers, a checklist will be completed to gather information on the characteristics of the various healthcare facilities and to maintain a record of these characteristics.

The list consists of the following items:

1. Total number of staff
2. Total number of nursing staff
3. Total number of doctors
4. Total number of doctors treating patients with dementia
5. Total number of patients attended per year
6. Availability of a pharmacy
7. Availability of a laboratory (number of operating days per week)
8. Health promotion activities related to dementia
9. Educational activities related to dementia for caregivers and patients

I.1.2. List of Actors to Recruit, Quotas, and Inclusion Criteria

The study will aim to gather opinions from a diverse range of perspectives, particularly from individuals familiar with the healthcare system's operations. In the case of healthcare personnel, such as directors, staff members, and pharmacy managers, it will be essential to interview those with extensive experience in their region and within the healthcare system. Given the high turnover rates in the healthcare sector, it may be pertinent to interview individuals other than the current officeholders.

For example, if the director of a healthcare facility has recently been replaced and a new individual has only been in the position for a week, it is likely that the new director may not yet be fully acquainted with the operational dynamics of the facility. In such cases, interviewing a more experienced person within the healthcare establishment may be advisable.

The following details will outline the inclusion criteria for the actors to be interviewed and the quota of interviews per type of actor.

I.1.3. Recruitment SOLELY FOR PATIENTS (Sub Study 1 and 2): Instructions: Pfeffer Functional Activity Questionnaire

For interviews with persons with dementia (PWD), the Pfeffer Functional Activity Questionnaire will be administered prior to conducting the interview. This assessment will aid in evaluating the functional status (degree of dependency in

daily life) of an individual and identifying participants with moderate or severe impairment who do not meet the established inclusion criteria.

The questionnaire will be administered to a close family member or the primary caregiver. The application will take approximately 5 to 7 minutes and will involve the following steps:

Processes:

- Engage with the caregiver or family member of the PWD.
- Explain the purpose of the project and the objective of the interview.
- Describe the Pfeffer test procedure.
- If a score of 6 or higher is obtained, the interview with the PWD will not be conducted. However, an interview with the caregiver or family member may proceed if permitted.
- If a score of 5 or lower is obtained, informed consent will be obtained from the caregiver, along with assent from the PWD.

General Characteristics of the Pfeffer Test:

- The test consists of 11 questions.
- Responses will be provided by an informant (close family member or primary caregiver).
- For this study, scoring will be primarily based on the individual's cognitive ability. This means that if the person has any physical limitations requiring assistance with the activities mentioned in the test but is mentally capable of executing the cognitive processes (reasoning, planning, decision-making, etc.), they will be rated as normal.
- Each item will be scored from 0 to 3:
    - 0: normal or never did it but could do it alone.
    - 1: with difficulty, but manages independently, or never did it and would have difficulty doing it alone.
    - 2: requires assistance but can perform the activity.
    - 3: dependent, cannot perform the activity.
- **Total Score:**
    - This is obtained by summing the scores for each item (ranging from 0 to 33 points).

    - A higher score indicates greater dependency.
- **Result Interpretation:**
    - A score below 6 (≤ 5) indicates normal functioning (no dependency).
    - A score of 6 or higher indicates functional impairment (functional dependency).

I.1.4. Guidelines for Presenting the Pfeffer Scale Questionnaire to Caregivers

These guidelines aim to clarify the purposes and characteristics of the Pfeffer Scale assessment, which measures the level of cognitive impairment in individuals. Specifically, these guidelines seek to reduce or avoid any misinterpretation of the questionnaire's objectives that may influence the responses and invalidate the final score.

The questionnaire should not be understood as:

- An evaluation of the caregiver's performance.
- An opportunity for the caregiver to "punish" or "reward" the person they care for.
- A means to obtain any benefit.

The questionnaire should be viewed solely as a list of questions designed to assess the autonomy and functionality of the individual in their daily routine.

1) Recommendations for Initial Contact with the Family

- Initiate contact with the caregiver to explain the upcoming procedures, allowing them to communicate the information to the person they care for.
- Inquire about the best setting for conducting the interview.
- Avoid mentioning the terms “inclusion criteria” or “exclusion criteria,” as these may be negatively interpreted.
- Remind the caregiver that they can be interviewed regardless of the test results.

2) Presentation Script "Good morning. As we discussed over the phone, we are conducting research on how the healthcare system addresses the needs of individuals with dementia. We would like to interview you, the caregiver, as well as the person you care for. To determine if we can interview them, we will need to ask you some questions to assess their suitability. This is a questionnaire directed at you, where we will ask simple questions about the daily activities of the person you care for. Please do not interpret this as an evaluation of you, and try to respond as

realistically as possible. If you have any questions about the questionnaire, feel free to ask, and we will clarify."

I.1.5. Step-by-Step Recruitment Process

The team will coordinate to schedule interviews with the various actors to be interviewed in the study. The following steps should be followed to recruit to the interviews:

Coordination with Healthcare Facilities (hospitals, community mental health centers, or health centers) to Interview Healthcare Personnel:

1. Selection of participating healthcare facilities will involve mapping potential community mental health centers, health centers, and hospitals.
2. Presentation of the project to the institution's director [assess if they are the appropriate person for interviews; if so, schedule an interview day or take advantage of the moment if they are available]. In some cases, research protocols may need to be submitted for review by their ethics committees.
3. Obtain authorization to conduct interviews with healthcare personnel.
4. Speak with potential interviewees to check their interest in, availability for, participating in the interviews.
5. Schedule the date and time for the interview [if the person is free and wishes to, conduct the interview straight away]. Request a mobile number to send a reminder about the interview.
6. Record the date and time in a link provided.
7. Attend the scheduled date and time.

Coordination’s Outside Healthcare Facilities (community health agents, patients, caregivers):

1. Coordinate with healthcare facilities to facilitate contact with community agents actively engaged in their communities.
2. Schedule an interview with the community agents to inquire if they are aware of cases of individuals with mild cognitive decline in their area.
3. Coordinate with the community agents to introduce them to the individuals with cognitive decline or their family members.
4. Once the individuals with cognitive decline have been identified, converse with their caregiver and administer the Pfeffer test.
5. Record the date and time in the link

IMPORTANT: Ensure that the scheduled interview date and time allow the interviewee sufficient time to complete the interview with minimal interruptions.

Interviews with Caregivers and Individuals with Cognitive Decline

The primary point of contact will be the community health agent, who will facilitate contact with the caregiver or members of the household of the individual with cognitive decline. Once introduced, the Pfeffer test should be administered to the caregiver to determine if the individual with cognitive decline can be interviewed. If the Pfeffer test score is below 6, the caregiver and the individual with cognitive decline will be invited to participate in the interviews.
