# Supplementary material for: Assessment of Health System Readiness and Quality of Dementia Services in Peru: Protocol for a Qualitative Study With Stakeholder Interviews and Documentation Review
Source: JMIR Res Protoc. 2025 Mar 21;14:e60296. doi: 10.2196/60296 (PMC11971575; doi:10.2196/60296)
Supplement: Multimedia Appendix 2 [file resprot_v14i1e60296_app2.pdf]

IMPACT Dementia: Health Systems Readiness Assessment
QUESTIONNAIRE: HEALTH WORKER

INTERVIEW INFORMATION:

Nº	Question	Answer
a	Code of interview	☐☐☐☐☐☐☐☐<br>Zone - Stakeholder - Interviewer - # questionnaire
b	Name or code of interviewer	☐☐
c	Date of interview (dd/mm/yyyy)	☐☐/☐☐/☐☐☐☐
d	Location of interview	
e	Name of the organisation/institution	
f	Observations	

1. General information General information about the site in which the stakeholder works and their job role and experience/background.

№	Question	Answer
1.1	Type of facility	1.Health post ( )
		2. Health centre ( )
		3. Public hospital ( )
		4. Private clinic ( )
		5. Independent/private ( )
		6. Other (specify) ( ):
1.2	Level of facility	
1.3	Sector where facility belongs to	1. Ministry of Health ( )
		2. Social security ( )
		3. Forced Army ( )
		4. Private sector ( )
		5. Other (specify)( ):
1.4	Area where the interviewee works	
1.5	Sex	1. Female ( )
		2. Male ( )
1.6	Occupational category	1. Specialist (specify) ( ):
		2. General practitioner ( )
		3. Nursing Staff (specify)( ):
		4. Therapist (specify)( ):
		4. Other (specify)( ) :
1.7	Years working in his/her current role / in health care?	
1.8	Job role description	

2. Healthcare structure and organisation of the health system:

№	Question	Answer
2.1	Within your workplace, is there a **unit/branch/department** responsible for **dementia** cases?<br><br>**If yes**, what is its name and who is in charge of it?<br>**If no,** is there any other organisation/ health facility to which dementia cases are referred to?	YES( ) NO( ) DO NOT KNOW( )<br>Comment:

2.2	How **well prepared** is your healthcare facility **to provide health care services for dementia** in terms of (a) the **staff and** (b) **physical/material resources** needed?	(a)Staff:<br><br>(b)Physical / Material resources:
2.3	How **well prepared** is your healthcare facility to provide **health care services to patients with comorbidities** in terms of (a) the staff and (b) physical/material resources needed?	(a)Staff:<br><br>(b)Physical / Material resources:
2.4	What (a) **other services** are available **in the community** to **support** management of **dementia**, and (b) how can patients **access them**?	(a)Services available:<br><br>(b)How can patients access the services:
2.5	Is there an **association, group or club for patients with dementia** connected to this facility? (non-professional associations). Please explain	YES ( ) NO ( ) DO NOT KNOW( )<br>Explain:

3.Policy environment:

Nº	Question	Answer
3.1	Do you know the **policies, programs or plan**s that exist at a national, regional or local level **for patients with dementia**?<br>**If yes,** list them.	YES ( ) NO ( ) DO NOT KNOW( )<br>**If yes,** list them:
3.2	In your opinion, how **supportive** is the **policy environment** (policies, programs or plans) related to dementia?<br><br>**Rate** from 1 to 5 (circle a number), where 1 is a weak	Rate: (Weakest support) 1 2 3 4 5 (Strongest support)<br><br>Explain:

	support and 5 Is a strong support. **Explain.**	
3.3	In your opinion, have the existing **policies, programs or plans** been **well implemented**? Explain.	YES ( ) NO ( ) DO NOT KNOW( )<br>Explain:
3.4	In your opinion, are there any aspects to improve in existing policies/programmes/plans for dementia? Explain.	YES ( ) NO ( ) DO NOT KNOW( )<br>Explain:

4. Financing:

№	Question	Answer
4.1	Are there any specific **barriers or difficulties** related to the **funding** devoted to **prevention, diagnosis and management of patients with dementia**?	
4.2	Do you think that the **available funding for prevention, diagnosis and management of dementia is adequate**? Explain.	YES ( ) NO ( ) DO NOT KNOW( )<br>Explain:
4.3	Do you think the **funding** for the **social or health protection** of the **elderly** is adequate? Explain	YES ( ) NO ( ) DO NOT KNOW( )<br>Explain:
4.4	Do you know if any **financial support** is available **for patients with dementia** and their **caregivers**? Comment.<br><br>**If no,** go to the next section	YES ( ) NO ( ) DO NOT KNOW( )<br>Comment:
4.5	What is the **process for accessing financial support**	(a)People diagnosed with dementia:

	(e.g. monetary transfer) for (a) **people with dementia**?<br><br>What is the **process for** (b) **someone who does not know if they have access to financial support**, or (c) if they are not currently accessing health insurance?	(b)People who doesn't know it they have access to financial support:<br><br>(c) People without access to health insurance:

5. Data collection and information systems:

№	Question	Answer
5.1	Does a **special registry** for recording **cases of dementia** exist in this facility?<br><br>**If yes**, how well does this registry work?	YES ( ) NO ( ) DO NOT KNOW ( )<br>**If yes,**<br>How well it works:
5.2	For this facility, do you know if any **statistical information** is **available** on:<br>(a) **Morbidity**: how many patients are there with dementia in a year and<br><br>(b) **Use of services**: number consultations in a year per dementia patient?	(a) Morbidity:<br><br>(1) Is there?: YES ( ) NO ( ) DO NOT KNOW ( )<br><br>(2) Accessible?: YES ( ) NO ( ) DO NOT KNOW ( )<br><br>Comment:<br><br>(a) Use of services:<br><br>(1) Is there?: YES ( ) NO ( ) DO NOT KNOW ( )<br><br>(2) Accessible?: YES ( ) NO ( ) DO NOT KNOW ( )<br><br>Comment:

6. Availability/Affordability of medications and tests:

№	Question	Answer
6.1	Does your (Country/Region) have a **list of essential diagnostic tests?** Comment.	YES ( ) NO ( ) DO NOT KNOW ( )<br>Comment:

6.2	Do you know what **diagnostic tests for dementia** are actually present **in this facility**?<br><br>**If yes**, list them.<br><br>Collect specific information about the use of each test:<br><br>(a) What is your **opinion** about them?<br><br>(b) **Problems or difficulties** to apply them.	YES ( ) NO ( ) DO NOT KNOW ( )<br><br>**If yes,** list them:<br>Mini-Mental State Examination MMSE ( )<br>Clock Drawing Test ( )<br>Montreal Cognitive Assessment (MoCA) ( )<br>Other (specify)( ):<br><br>(a)Opinion about them:<br><br>(b) Problems or difficulties to apply them:
6.3	In your daily practice, what t**ype of drugs do you use** for people with **dementia**?<br><br>e.g: antipsychotics, antidepressant	
6.4	Does your (Country/Region) have an essential medicines list for dementia? **If yes,** list and comment about the.	YES ( ) NO ( ) DO NOT KNOW ( )<br>**If yes,**<br>List them:<br>Donepezil ( )<br>Rivagstigmine ( )<br>Galantamine ( )<br>Memantine ( )<br><br>Comments:
6.5	Do you know what **medicines for dementia** are actually **present** in this **facility**? **If yes**, list them.	YES ( ) NO ( ) DO NOT KNOW ( )<br>**If yes**,<br>Comment :
6.6	What **problems or difficulties** do **patients with dementia** often experience in having:<br>(a) regular access **products/technologies** needed for **diagnosis**,<br>(b) having a **regular supply** of **medicines**?	(a) Regular access to products/technologies for diagnosis<br><br>(b) Regular supply of medicines

6.7	What **problems or difficulties** do **patients with dementia and comorbidities** often experience in having:<br>(a) Regular access **products/technologies** needed for **diagnosis**<br><br>(b) having a **regular supply** of **medicines**?	(a) Regular access to products/technologies for diagnosis<br><br>(b) Regular supply of medicines

7. Barriers to diagnosis and treatment:

Nº	Question	Answer
7.1	What are the (a) main **problems or difficulties** you face regarding the **diagnosis process of dementia**? (b) How **frequently** do they occur?	(a) Problems:<br><br>(b)Frequency of occurrence:
7.2	What are the (a) main **problems or difficulties** you face in providing **adequate attention** to patients with **dementia**? (b) How **frequently** do they occur?	(a) Problems<br><br>(b)Frequency of occurrence:
7.3	Based on your experience, what is the **hardest part of dementia care** for (a) **patients** and (b) their **caregivers**?	(a)Patients:<br><br>(b)Caregivers:
7.4	Do you think (a) **patients and** (b) their **caregivers have enough knowledge** about dementia? Explain your answer.	YES ( ) NO ( ) NOT ANSWER ( )<br>(a) Patients:<br><br>(b) Caregivers:

7.5	What are the main **factors** that **affect** (a) patient and (b) caregiver **education** in cases of **dementia**?	(a) Patients:<br><br>(b) Caregivers:
7.6	What **problems or difficulties** do you believe **patients with dementia** experience with **self-management**?	
7.7.	What are the main **problems or difficulties** you face in providing adequate attention **to patients with dementia and comorbidities** (e.g. diabetes, hipertension, others)?	

8. Service delivery in prevention and management issues:

№	Question	Answer
8.1	Does this facility have any **guidelines** in case of **warning signs** of a **possible diagnosis of dementia** in a patient? **If yes**, explain.	YES ( ) NO ( ) NOT ANSWER ( )<br>**If yes,**<br>Explain:
8.2	Does this facility have any **guidelines** to **manage dementia** cases? **If yes**, explain.	YES ( ) NO ( ) NOT ANSWER ( )<br>**If yes,**<br>Explain:
8.3	What are the main problems regarding **service delivery for dementia** in the **Primary Health Care level** of attention?	
8.4	What are the **main problems regarding service delivery** for dementia in (a) **the secondary** and (b) **tertiary levels** of attention?	(a) Secondary level:<br><br>(b) Tertiary level:

8.6	What **documents** do you provide to **facilitate** the **care of the person with dementia** (prescriptions, instructions on follow-up visits, etc.)?	
8.7	In this facility, do **patients with dementia receive information** about healthy diet, physical activity and other advice related to **prevention of dementia**?<br><br>**If Yes**, what type of information do they receive and in which format (e.g. interpersonal communication with a health worker, brochure, referral to a website)?	YES ( ) NO ( ) DO NOT KNOW ( )<br><br>**If yes,**<br>(a)Type of information:<br><br>(b)Format of information:
8.7	In your facility, are there **informational campaigns for dementia** (note: could be for the general public or patients)? **If Yes**, what do they consist of?	YES ( ) NO ( ) DO NOT KNOW ( )<br>**If yes,**<br>Explain:
8.8	What other **information or training** is **provided to** (a) **patients with dementia** and their (b) **caregivers**?	(a) Patients:<br><br>(b) Caregivers:
8.9	In your opinion, **who demands more healthcare services** for dementia, **female or male**? Why?	

9. Training/capacity to provide care:.

Nº	Question	Answer
9.1	In this facility, are **educational classes or training** provided for professionals about **dementia**? Explain.	YES ( ) NO ( ) DO NOT KNOW ( )<br>Explain:
9.2	Have you received **special training** for attending **patients with dementia**?<br><br>**If Yes**, please indicate what training ,where, when, what frequency, who carried out the training.	YES ( ) NO ( ) DO NOT KNOW ( )<br>**If yes,**<br>Explain:
9.3	Do you believe that your **training is sufficient to treat patients with dementia**? Explain.	YES ( ) NO ( ) DO NOT KNOW ( )<br>Explain:
9.4	Have you received **training for orienting caregivers of patients** with dementia?<br><br>**If Yes**, explain (what training, where, when, what frequency, who carried out the training).	YES ( ) NO ( ) DO NOT KNOW ( )<br>**If yes,**<br>Explain:
9.4	Which **topics about dementia** do you need to **improve?**<br><br>*Ask about prevention, diagnosis, delivery of care?	
9.6	Is your **work with patients with dementia supervised**? **If Yes**, by whom?	YES ( ) NO ( ) DO NOT KNOW ( )<br>**If Yes,** by whom?:
9.7	In your region, are there any **guidelines or protocols** being recommended or used **for dementia cases**?<br><br>**If Yes**, (a) what type? (b)Is it useful for your work with these patients?	YES ( ) NO ( ) DO NOT KNOW ( )<br>**If yes,**<br><br>(a) Type of protocols:<br><br>(b)Usefulness:

10. Medical technologies and infrastructure.

Nº	Question	Answer
10.1	Do you know about any **health care service using a mobile device in your facility?** (e.g., smartphone, tablet)?<br><br>**If yes**,<br>(a) what is it used for? (b)do you know how well it works?<br>(c) What are the barriers and (d)facilitators to its use within the facility? (e) Do PWD and their carers use this service?	YES ( ) NO ( ) DO NOT KNOW ( )<br><br>**If yes**:<br>(a)How is it used for:<br><br>(b)How well it works:<br><br>(c)Barriers:<br><br>(d)Facilitators:<br><br>(e)Usage by PWD and carers:
10.2	Do you have **access to a mobile device** (e.g., smartphone, tablet) for work?<br><br>**If yes**, (a)does it have access to the internet? (b) Do you use it for work? What do you use it for? (e.g., contact with patients, registration of patient's data, treatment)	YES ( ) NO ( ) DO NOT KNOW ( )<br>**If yes:**<br>(a) Access to internet:<br><br>(b) Use for work:<br><br>(c) Frequency of use:

11.Perceptions of and experience with using mHealth technology:

Nº	Pregunta	Respuesta
11.1	Do you know what **tools are currently used in the health system for dementia screening?** If yes, please provide more details.	YES ( ) NO ( ) NOT ANSWER ( )<br>Comment:
11.2	¿**If** you had **all the resources needed**, what **intervention to increase dementia detection** would you propose for the country?	

11.3	**Considering the actual available resources** in the health system:<br>(a) What **intervention to increase dementia detection** would you propose for the country?<br>(b) **Who** will be **in charge** of it?<br>(c) **How** will this **diagnostic intervention be implemented**?	(a) intervention to increase dementia detection:<br><br>(b) Who will be in charge:<br><br>(c) How will it be implemented:
11.4	Do you know what **tools are currently used** in the health system **for dementia management**? If yes, please provide more details.	YES ( ) NO ( ) NOT ANSWER ( )<br>**If yes,**<br>Comment:
11.5	**If** you had **all the resources needed**, what **intervention for dementia management** would you propose for the country?	
11.6	**Considering the actual available resources** in the health system:<br>(d) What **intervention for dementia management** would you propose for the country?<br>(e) **Who** will be **in charge** of it?<br>(f) **How** will this **diagnostic intervention be implemented**?	(d) intervention for dementia management:<br><br>(e) Who will be in charge:<br><br>(f) How will it be implemented:
11.7	Do you know of **applications on mobile devices** (smartphones, tablets) that perform **diagnostic or disease management interventions**?<br><br>**If yes,** comment on their most important features (disease targeted, functions, advantages, disadvantages).<br><br>**If not,** what would you think if mobile devices (smartphones, tablets) were used for diagnosis or disease management?	YES ( ) NO ( ) NOT ANSWER ( )<br><br>**If yes,**<br>Comment:<br><br>**If not,**<br>Comment:

11.8	On **<u>mobile health interventions to detect or manage cases</u>** (health care using applications on mobile phones or tablets) **<u>of people diagnosed with dementia</u>**:<br><br>(a) What benefits do you think they could have?<br><br>(b) What difficulties or problems do you think they could bring?<br><br>(c) Considering your knowledge of the health care system, do you think this type of service could be implemented effectively?	(a) Benefits:<br><br>(b) Difficulties or problems:<br><br>(c) Do you think this type of service could be implemented effectively?:
11.9	What do you think about an **intervention using applications on mobile devices** (smartphones or tablets) **in which validated dementia screening questionnaires are answered?**<br><br>(a) Comment on the pros and cons you can identify.<br><br>(b)If this intervention is developed, which actor in the health system do you think should apply the questionnaires presented in the app to users (e.g., doctors, nursing technicians)?<br><br>**(c) If CHW are not mentioned**, ask: What do you think about having a CHW to implement it?	(a) Pros and cons:<br><br>(b) Stakeholder that should apply questionnaires on the app:<br><br>(c) If CHW is not mentioned, ask for opinion:
11.10	¿Qué opina de una **intervención en la que el manejo de la demencia** (información sobre la enfermedad, seguimiento de síntomas, monitoreo de citas) **se brinde a pacientes y cuidadores usando tecnología móvil** (con un aplicativo en un teléfono inteligente o tableta) Comente los aspectos a favor y en contra que pueda identificar.	(a) Pros<br><br>(b) Cons

IMPACT Dementia: Health Systems Readiness Assessment

QUESTIONNAIRE: PATIENT/CAREGIVER

INTERVIEW INFORMATION:

Nº	Pregunta	Respuesta
a	Interview code	☐☐☐☐☐☐☐☐<br>Zone - Stakeholder - Interviewer - # questionnaire
b	Interviewer code	☐☐
c	Date of the interview (dd/mm/yyyy)	☐☐/☐☐/☐☐☐☐
d	Interview location	
e	Observations	

1.General information:

Nº	Question	Answer
1.1	**Patient:** Patient category	1. Ambulatory ( )
		2. Community patient (outside of a facility) ( )
		3. Other (specify)( ):
1.2	**Patient:** Sex	Female ( ) Male ( )
1.3	**Patient:** Age	
1.4	**Patient:**Educational level	1. No education
		2. Primary school incomplete
		3. Primary school complete
		4. High school incomplete
		5. High school complete
		6. Higher education incomplete
		7. Higher education complete
		8. Other (specify)
1.5	**Patient:**Main occupation	
1.6	**Patient:** Which type of dementia have you been diagnosed with? (if known)	
1.7	**Patient:** what age were you diagnosed with dementia?	
1.8	**Patient:** What other illnesses or complications do you have?	
1.9	**Patient:** Where do you usually receive health care?<br><br>*Interviewee can select more than one option	1. SIS ( )
		2. EsSalud ( )
		3. Private insurance ( )
		4. Other (specify): ( )
1.10	**Patient:** Patient category	1. Ambulatory ( )
		2. Community patient (outside of a facility) ( )
		3. Other (specify)( ):
1.11	**Patient:** In which health facility do you receive regular health care?<br><br>*Interviewee can select more than one option	1. Hospital ( )
		2. Health post ( )
		3. Health center ( )
		4. Private clinic ( )
		5. Other (specify)( ):
1.11	**Caregiver:** Caregiver category	1. Family member (specify) ( ):
		2. Not a family member (specify) ( ):
1.12	**Caregiver:** Sex	
1.13	**Caregiver:** Age	
1.14	**Caregiver:**Educational level	1. No education

		2. Primary school incomplete
		3. Primary school complete
		4. High school incomplete
		5. High school complete
		6. Higher education incomplete
		7. Higher education complete
		8. Other (specify)
1.14	**Caregiver:** Additional occupation to caregiving role	

2. Healthcare structure and organisation of the health system:

№	Question	Answer
2. 1	**Patient: How well** does **the health system** where you receive care **respond to your needs** as a **patient with dementia**?<br><br>**Rate** from 1 to 5 (circle the number), where 1 is too bad and 5 is very well.<br><br>Please, comment on your answer.	Rate: (Too Bad) 1 2 3 4 5 (Very well)<br><br>Comment:
2. 2	**Caregiver: How well** does **the health system** where you receive care **respond to your needs as a caregiver of a patient with dementia**?<br><br>**Rate** from 1 to 5 (circle the number), where 1 is bad and 5 is very well.<br><br>Please, comment on your answer.	Rate: (Too Bad) 1 2 3 4 5 (Very well)<br><br>Comment:

3.Policy environment:

№	Question	Answer
3. 3	In your opinion, **how supportive is the policy environment (laws, regulations, plans)** for policies related **to dementia**?<br><br>**Rate** from 1 to 5 (circle a number), where 1 is a weak support and 5 is a strong support.<br><br>Please, explain your answer.	Rate: (Weakest support) 1 2 3 4 5 (Strongest support)<br><br>Explain:

4. Financing:

Nº	Question	Answer
4. 1	Do you have **health insurance**?<br><br>**If yes**,<br>(a) what type of health insurance do you have<br><br>(b)How **good or bad is its coverage** for **dementia** and other **comorbidities**?<br><br>(c) What **services** are **covered** by your health insurance?<br><br>(d) What **services are not covered** by your health insurance?<br><br>(e) How do you **pay for the services** that are **not covered** by your health insurance?<br><br>(f) Besides health services, are there any **other expenses you usually make to take care of your illness that are not covered** by your health insurance? List tem.<br><br>(g) Explain **how you pay** for those **extra expenses** not covered by your health insurance.<br><br>**If not**, go to question 4.2	YES ( ) NO ( ) DO NOT KNOW ( ) NOT ANSWER ( )<br><br>**If Yes**,<br>(a) Type of health insurance:<br><br>(b) How good or bad is the coverage:<br><br>(c) Services covered:<br><br>(d) Services not covered:<br><br>(e) How not covered services are paid:<br><br>(f) Other expenses not covered by health insurance:<br><br>(g) How other expenses are paid:

4.2	Can you tell us (a) **why you don't have health insurance**?<br><br>(b) How do you **pay for the expenses** of your disease?	(a) why don't have health insurance:<br><br>(b) How the expenses of your disease are paid:
4.2	At any point, **has (a) taking care for dementia created financial problems**? **If yes**, explain your answer.<br><br>(b) Has taking **care of other illnesses created financial problems**? **If yes**, explain your answer.	YES ( ) NO ( ) DO NOT KNOW ( ) NOT ANSWER ( )<br>**If yes, financial problems created by:**<br>(a) Dementia:<br><br>(b) Other illnesses:
4.3	At any point, **have you had to stop following** the instructions of the doctor for **treatment for dementia due to financial difficulties**?<br><br>**If yes,** (a) **explain** your answer and (b) **what part of the treatment you had to stop** completely or partially.<br><br>*Interviewee can select more than one option.	YES ( ) NO ( ) DO NOT KNOW ( ) NOT ANSWER ( )<br>**If yes,**<br>(a) Explain:<br><br>(b) Part of treatment that had to stop:<br>1. Medication ( )<br>2. Laboratory tests or exams ( )<br>3. Follow-up check-ups (monitoring) ( )<br>4. Hospitalisation ( )<br>5. Diet ( )<br>6. Physical activity ( )<br>7. Rehabilitation ( )<br>8. Other (specify) ( ):
4.4	At any point, **have you had to stop following** the instructions of the doctor for **treatment for other illnesses due to financial difficulties**?<br><br>**If yes,** (a) **explain** your answer and (b) **what part of the treatment you had to stop** completely or partially.<br><br>*Interviewee can select more than one option.	YES ( ) NO ( ) DO NOT KNOW ( ) NOT ANSWER ( )<br>**If yes,**<br>(a) Explain:<br><br>(b) Part of treatment that had to stop:<br>1. Medication ( )<br>2. Laboratory tests or exams ( )<br>3. Follow-up check-ups (monitoring) ( )<br>4. Hospitalisation ( )<br>5. Diet ( )<br>6. Physical activity ( )<br>7. Rehabilitation ( )<br>8. Other (specify) ( ):

4.6	Is the **family budget** also used to **pay for the expenses** of your disease? Explain your answer.	YES ( ) NO ( ) DO NOT KNOW ( ) NOT ANSWER ( )<br>**If yes,**<br>Explain:

5. Data collection and information systems:

Nº	Question	Answer
5.1	Have you ever had problems with your medical records at the health facility you usually go to? **If yes**, please explain the problems.	YES ( ) NO ( ) DO NOT KNOW ( ) NOT ANSWER ( )<br>**If Yes,**<br>Explain:

6. Availability/Affordability of medications and tests:

Nº	Question	Answer
6.1	During the diagnosis process for dementia, **did you have access to the test/medical resources needed**? Comment on your answer.	YES ( ) NO ( ) DO NOT KNOW ( ) NOT ANSWER ( )<br>Comment:
6.2	Do you **receive any treatment** for **dementia**? **If yes,** (a) what treatment or instructions for controlling it have you been prescribed by a healthcare professional?<br><br>(b) What type of professional gave the instructions?<br><br>**If not, why** don't you receive treatment for **dementia**?	YES ( ) NO ( ) DO NOT KNOW ( )<br>**If yes,**<br>(a) Treatment details:<br><br>(b) Professional that gave the treatment:<br><br>**If not,** why?:
6.3	Do you **receive any treatment for other illnesses**?<br><br>**If yes,** what treatment or instructions for controlling them have you been prescribed by a healthcare professional?	YES ( ) NO ( ) NOT ANSWER ( )<br>**If yes,**<br>Treatment details:

6.4	Please, **describe the pharmacological treatment for other illnesses prescribed to you** (and dosage).	
6.5	Do you **adhere to treatment** and why (explore difficulties)?<br><br>(a)Take all **medicines**<br><br>(b)Follow **schedules**<br><br>(c) Follow **lifestyle recommendations**<br><br>*Specify if interviewee mention that treatment was not prescribed	YES ( ) NO ( ) NOT ANSWER ( )<br><br>(a) Take all medicines: YES ( ) NO ( ) NOT ANSWER ( )<br><br>Why (explore difficulties):<br><br>(b) Follow schedules: YES ( ) NO ( ) NOT ANSWER ( )<br><br>Why (explore difficulties):<br><br>(c)Follow lifestyle recommendations<br>YES ( ) NO ( ) NOT ANSWER ( )<br><br>Why (explore difficulties):
6.6	**If the interviewee uses medication: Where do you obtain** (or buy) your medications?<br><br>*Interviewee can select more than one option	1. Pharmacy of the facility where usually goes ( )
		2. The pharmacy of other public facility ( )
		3. The pharmacy of a private facility ( )
		4. An external private pharmacy ( )
		5. Other (specify) ( ):
6.7	**Only if the person buys their medication outside of the facility:** Why don't you get your medication within this facility?	

7. Barriers to diagnosis and treatment:

Nº	Question	Answer
7.1	Do you remember **in what place or facility you were first diagnosed with dementia**?	1. Health post
		2. Health centre

		3. Hospital<br>4. Clinic/private office<br>5. Community: screening or screening campaign<br>6. Do not know/ refuse to answer<br>7. Other (specify):
7.2	Is it **the same place you are being treated**?<br><br>**If No,** where are you currently receiving treatment?	YES ( ) NO ( ) NOT ANSWER ( )<br><br>**If No,**<br>where is receiving treatment:
7.3	Have you been hospitalised due to complications of this disease?<br><br>**If yes,**<br>(a) **where and when** were you hospitalised?<br><br>(b) Do you remember if you **had any problems or delays to receive healthcare attention** when hospitalised?	YES ( ) NO ( ) NOT ANSWER ( )<br><br>**If yes,**<br><br>(a)where and when:<br><br>(b)Problems/delays:
7.5	Do you think **health professionals have enough knowledge** about dementia? Explain your answer.	YES ( ) NO ( ) NOT ANSWER ( )<br>Explain:

8. Service delivery in prevention and management issues:

Nº	Question	Answer
8.1	**Patient:** Have you **received information about prevention, symptomatology and treatment for dementia**?<br><br>**If yes**, (a) **describe** the information (in which format and who gave the information) and<br><br>(b) Did you find it **useful**?	YES ( ) NO ( ) DO NOT KNOW ( )<br><br>**If yes:**<br>(a)Description of information received:<br><br>(b)Usefulness
8.2	**Patient:** Have you **received information about prevention, symptomatology and treatment for other illnesses**?	YES ( ) NO ( ) DO NOT KNOW ( )<br><br>**If yes:**

	**If yes**, (a) **describe** the information (in which format and who gave the information) and<br><br>(b) Did you find it **useful**?	(a)Description of information received:<br><br>(b)Usefulness
8.3	**Caregiver:** Have you received **information about how to support and take care of the person you are caring fo**r?<br><br>**If yes**, (a) **describe** the information (in which format and who gave the information) and<br><br>(b) Did you find it **useful**?	YES ( ) NO ( ) DO NOT KNOW ( )<br><br>**If yes:**<br><br>(a)Description of the information:<br><br>(b)Usefulness
8.4	Have you ever been **referred from one health facility to another for treatment for dementia or due to a complication?** E.g. For laboratory tests, consultations, hospitalisation.<br><br>**If yes**, please specify where.	YES ( ) NO ( ) NOT ANSWER ( )<br><br>**If Yes,** where (referral)?:
8.5	Do you remember if there were a**ny problems getting a referral**? **If yes,** explain the problems	YES ( ) NO ( ) DO NOT KNOW ( )<br>Explain:
8.6	What are the **positive aspects of the treatment and management you receive for dementia**?	
8.7	What (a) **is missing in the treatment and management of their dementia**? (b) What **improvements** could be made?	(a)Missing aspects:<br><br>(b)Improvements:
8.8	In your opinion, what are the **main problems** regarding **service delivery for dementia in the Primary Health Care level of attention** (e.g. in your health post or primary centre)?	

8.9	In your opinion, what are the **main problems** regarding s**ervice delivery for dementia in the** (a) **secondary and** (b) **tertiary levels of attention** (hospitales)?	(a) Secondary level<br><br>(b) Tertiary level
8.10	Do you know about any **social or community services** available **for people with dementia**?<br><br>**If yes,** do you use them? Ask for more detail about the service the interviewee uses.	YES ( ) NO ( ) NOT ANSWER ( )<br><br>**If yes,** do you use it?:

9. Training/capacity to provide care:

Nº	Question	Answer
9. 1	Do you believe that the **healthcare staff** at the facilities you have received care **are sufficiently trained** to treat patients with **dementia?** Explain.	YES ( ) NO ( ) DO NOT KNOW ( )<br><br>Explain:
9. 2	Do you believe that the **healthcare staff** at the facilities you have received care **are sufficiently trained** to **orient and manage caregivers** of patients with dementia? Explain.	YES ( ) NO ( ) DO NOT KNOW ( )<br><br>Explain:

10. Medical technologies and infrastructure:

Nº	Question	Answer
10. 1	Do you have **access to a mobile device** (e.g., smartphone, tablet)?<br><br>**If yes**, (a) do you have access to the internet? (b)What do you use it for? (c) How frequently do you use it?	YES ( ) NO ( ) NOT ANSWER ( )<br><br>**If yes,**<br>(a) Access to internet:<br><br>(b)Uses:<br><br>(c) Frequency of use:

11.Perceptions of and experience with using mHealth technology:

Nº	Pregunta	Respuesta
11.1	Do you know what **tools are currently used in the health system for dementia screening?** If yes, please provide more details.	YES ( ) NO ( ) NOT ANSWER ( )<br>Comment:
11.2	Do you know what **tools are currently used** in the health system **for dementia management**? If yes, please provide more details.	YES ( ) NO ( ) NOT ANSWER ( )<br>**If yes,**<br>Comment:
11.3	Do you know of **applications on mobile devices** (smartphones, tablets) that perform **diagnostic or disease management interventions**?<br><br>**If yes,** comment on their most important features (disease targeted, functions, advantages, disadvantages).<br><br>**If not,** what would you think if mobile devices (smartphones, tablets) were used for diagnosis or disease management?	YES ( ) NO ( ) NOT ANSWER ( )<br><br>**If yes,**<br>Comment:<br><br>**If not,**<br>Comment:
11.4	On **mobile health interventions to detect or manage cases** (health care using applications on mobile phones or tablets) **of people diagnosed with dementia**:<br><br>(a) What benefits do you think they could have?<br><br>(b) What difficulties or problems do you think they could bring?<br><br>(c) Considering your knowledge of the health care system, do you think this type of service could be implemented effectively?	(a) Benefits:<br><br>(b) Difficulties or problems:<br><br>(c) Do you think this type of service could be implemented effectively?:
11.5	What do you think about an **intervention using applications on mobile devices** (smartphones or tablets) **in which validated dementia**	(a) Pros and cons:

	**screening questionnaires are answered?**<br><br>(a) Comment on the pros and cons you can identify.<br><br>(b)If this intervention is developed, which actor in the health system do you think should apply the questionnaires presented in the app to users (e.g., doctors, nursing technicians)?<br><br>**(c) If CHW are not mentioned**, ask: What do you think about having a CHW to implement it?	(b) Stakeholder that should apply questionnaires on the app:<br><br>(c) If CHW is not mentioned, ask for opinion:
11.6	What do you think of an intervention in which **dementia management** (information about the disease, symptom tracking, appointment monitoring) is provided to patients and carers **using mobile technology** (with an application on a smartphone or tablet)? Comment on the (a) pros and (b) cons you can identify.	(a) Pros<br><br>(b) Cons
