# Supplementary material for: Assessment of Health System Readiness and Quality of Dementia Services in Peru: Protocol for a Qualitative Study With Stakeholder Interviews and Documentation Review
Source: JMIR Res Protoc. 2025 Mar 21;14:e60296. doi: 10.2196/60296 (PMC11971575; doi:10.2196/60296)
Supplement: Multimedia Appendix 3 [file resprot_v14i1e60296_app3.pdf]

IMPACT Salud: Patient Journey Sub Study
INTERVIEW GUIDE

Specific Questions for the Caregiver

Guidelines for Proper Execution:

This is a semi-structured interview guide. Therefore, there are general questions that should encourage the interviewee to mention various aspects of a topic. Similarly, there are follow-up questions that point to essential topics that need to be addressed, and they can be used to delve deeper into areas if the interviewee has not already done so.

The objective of this guide is to gather information about the process of diagnosing and treating the disease.

The guide should not be seen as a script but rather as a tool for gathering information.

It is recommended to have one or more strategies for gathering this information. For example, you may start with topics the interviewee feels more comfortable discussing. Alternatively, instead of asking a general question, you could share a personal story that meets the same objective

Diagnostic Process

1. Good morning, this is an interview about the experience of obtaining a diagnosis and continuing or following dementia treatment in the Peruvian healthcare system. We would like to go step by step through your family member's story. For you, when does this story begin?
    - What were the first symptoms?
    - Was there any screening or early detection?
    - Did they already visit the doctor for other comorbidities?
    - What did the family think?
        - Was there any resistance to seeing a doctor to examine the symptoms?
    - What did they and their family think when they noticed the symptoms? Did they quickly think about seeking medical attention?
2. How was the process of obtaining the diagnosis?
    - How much time passed from the first appointment for symptoms to receiving the diagnosis? How many appointments were there?
    - Were they seen in a primary care facility? A secondary one?
    - How was that day? Do you remember the care received?

- How many sessions had there been before that?
- Do you remember how many people attended to them? Nurses, technicians, doctors?
- Was it through a screening test (mini-mental)? Blood tests? Imaging?
- Was it all three?
- How much time did it take to get an appointment for the tests?
- How long did you wait for the results?
- What information did the healthcare staff provide to you as a caregiver? What was your opinion about this information?
- What did you think about the care throughout the diagnostic process?

3. What did the family do when they learned about the diagnosis?
    - Was there a family meeting?
    - How were responsibilities resolved?
    - What comments were made? Was it a problem?
    - What actions were taken?

Disease Management Process

1. How was the care after the diagnosis?
    - What things changed?
    - What does the patient's routine consist of?
    - Did the household roles remain the same?
    - Did you feel prepared?
    - Were there any financial problems? How did you handle them?
    - Did you make any changes at home?
2. How were the doctor visits?
    - How often did or do you attend them?
    - How was the treatment during those visits?
    - Do you consider them useful?
    - Were any medications prescribed?
    - What medication do they take?
        - How do you obtain them?
    - What is your opinion about this?

3. What regular tasks are associated with the care you provide?
    - How did you learn to do them? Which of these tasks do you find most demanding?
    - What information has been most helpful in providing care to the person with dementia?
    - Where did you obtain this information?
    - Did you receive any documents/guidelines to facilitate the care of the person with dementia (prescriptions, instructions for follow-up visits, etc.)?
    - Is there any healthcare service that you find particularly useful for your caregiving activities?
    - Who provides it? Do you have to pay for it? In what specific ways is it helpful to you?

Comorbidities

1. What is the impact of comorbidities (e.g., cardiovascular diseases) on your family member's dementia?
    - On the diagnosis?
    - On the treatment?
    - On your caregiving burden?

Opportunities to Improve Dementia Diagnosis and Management

1. What recommendations can you offer to facilitate the diagnostic process?
2. What recommendations can you give to facilitate the treatment of dementia?
